# High-definition and low-noise muography of the Sakurajima volcano with gaseous tracking detectors

**DOI:** 10.1038/s41598-018-21423-9

**Published:** 2018-02-16

**Authors:** László Oláh, Hiroyuki K. M. Tanaka, Takao Ohminato, Dezső Varga

**Affiliations:** 1 Earthquake Research Institute, The University of Tokyo, 1-1-1 Yayoi, Bunkyo, Tokyo, 113-0032 Japan; 2 Wigner Research Centre for Physics of the Hungarian Academy of Sciences, 29-33 Konkoly-Thege Miklós Str., Budapest, H-1121 Hungary

## Abstract

Muography is a novel method to highly resolve the internal structure of active volcanoes by taking advantage of the cosmic muon’s strong penetration power. In this paper, we present the first high-definition image in the vicinity of craters of an erupting volcano called Sakurajima, Kyushu, Japan. The muography observation system based on the technique of multi-wire proportional chamber (mMOS) has been operated reliably during the data taking period of 157 days. The mMOS measured precisely the flux of muons up to the thickness of 5,000 meter-water-equivalent. It was shown that high-definition density maps around the Craters A, B and Showa could be determined with a precision of less than 7.5 × 7.5 m^2^ which earlier had not yet been achieved. The observed density distribution suggests that the fall back deposits filled the magma pathway and increased their density underneath Craters A and B.

## Introduction

Accurate measurements of the size and shape of the uppermost part of a given volcanic conduit provides useful information to us, in particular for modeling eruption dynamics; in fact, there is a large number of studies on shallow conduit flow processes and resultant lava dome behavior^1–3^. Melnik and Sparks explored how variations of conduit diameter or magma chamber crystal content controlled flow dynamics, and how large changes in discharge rate and eruptive behavior could occur as a consequence of small changes in conduit diameter along with magma temperature and water content^4^. Massol and Jaupart later studied the connections between conduit and dome flows, and specifically how eruption behavior depends on local flow conditions at the vent^5^. They identified that the magnitude of gas pressure at the vent is influenced by the shape of the shallow conduit beneath the vent.

Muography is a newly developed imaging technique utilising the high energy near-horizontally arriving cosmic muons and enabling us to create high-resolution images of the internal structure of a volcano^6–13^. Muons are continuously generated in the atmosphere as the secondary cosmic rays as a result of nuclear interactions between galactic cosmic rays and atmospheric nuclei such as oxygen and nitrogen. The open-sky near horizontal muon flux ranges from 0.5 to 10 m^−2^ sr^−1^ s^−1^ for arriving angles of 5–20 degrees from the horizon, and thus they can be used for imaging the temporal changes inside a volcano^10^. Although the targetable region is limited to the shallow part of a volcano, not like seismic tomography or electric resistivity measurements, a dense array with a large number of sensors is not required to create a high-definition image^14^. However, the resolving power of a successful volcano measurement has been up to 1.15° (20 mrad)^9^. This angular resolution would correspond to 60 m at a distance of 3 km that is not sufficient to measure the typical conduit diameter of 10 m. Higher resolution muography systems may be developed using gaseous detectors with improved resolution or using nuclear emulsions. For the former, one needs to consider using non-flammable gas mixtures for implementation of long term standalone field operation. The latter technique, i. e. nuclear emulsion, has the potential to create a high resolution image (better than 10 mrad). However this technique cannot be used for real-time monitoring.

Positional resolution at the target volcano depends on the distance between the observation system and the volcano. However, optimal placement of the muography observation system (MOS) is not always possible. Furthermore, even if the geographical features do not hamper the installation, governments often set up restricted zones around the perimeter of active volcanoes, typically 2 km from the active crater in Japan. Although Tanaka developed airborne muography^11^ that is not restricted by the geographical feature of the target volcano, a large scale drone instead of a man-operated helicopter will be required when the volcano is active. Therefore, an improved capability of higher angular resolution with lower background is required for practical muography.

In this work, we developed a high definition muography observation system for practical volcano monitoring based on the scintillator-based Muography Observation System (sMOS) that successfully imaged the sequential magma movement in Satsuma-Iwojima volcano^10^. By designing a system that takes advantage of multi-wire proportional chamber (MWPC) technology^15,16^ while also eliminating the necessity of using flammable gas, it becomes more practical for field works^16–18^. With this newly developed system, called MWPC-based Muography Observation System (mMOS), we generated a muographic image with the highest definition yet achieved in Sakurajima, Japan with sufficiently low background noise. The outline of the projection image was compared with the exterior shape of the mountain to confirm the current definition of our muographic image. Three small peaks at the top of the mountain were clearly resolved in the resultant muographic image, indicating the angular resolution is better than 3 mrad. The background rate was estimated by comparing between the measured and theoretically derived flux, and was confirmed to be less than 10^−2^ m^−2^ sr^−1^ s^−1^. The first high-definition muographic image of the internal structure of Sakurajima will be shown.

## Results

### Observation of Sakurajima volcano

**Figure 1:**
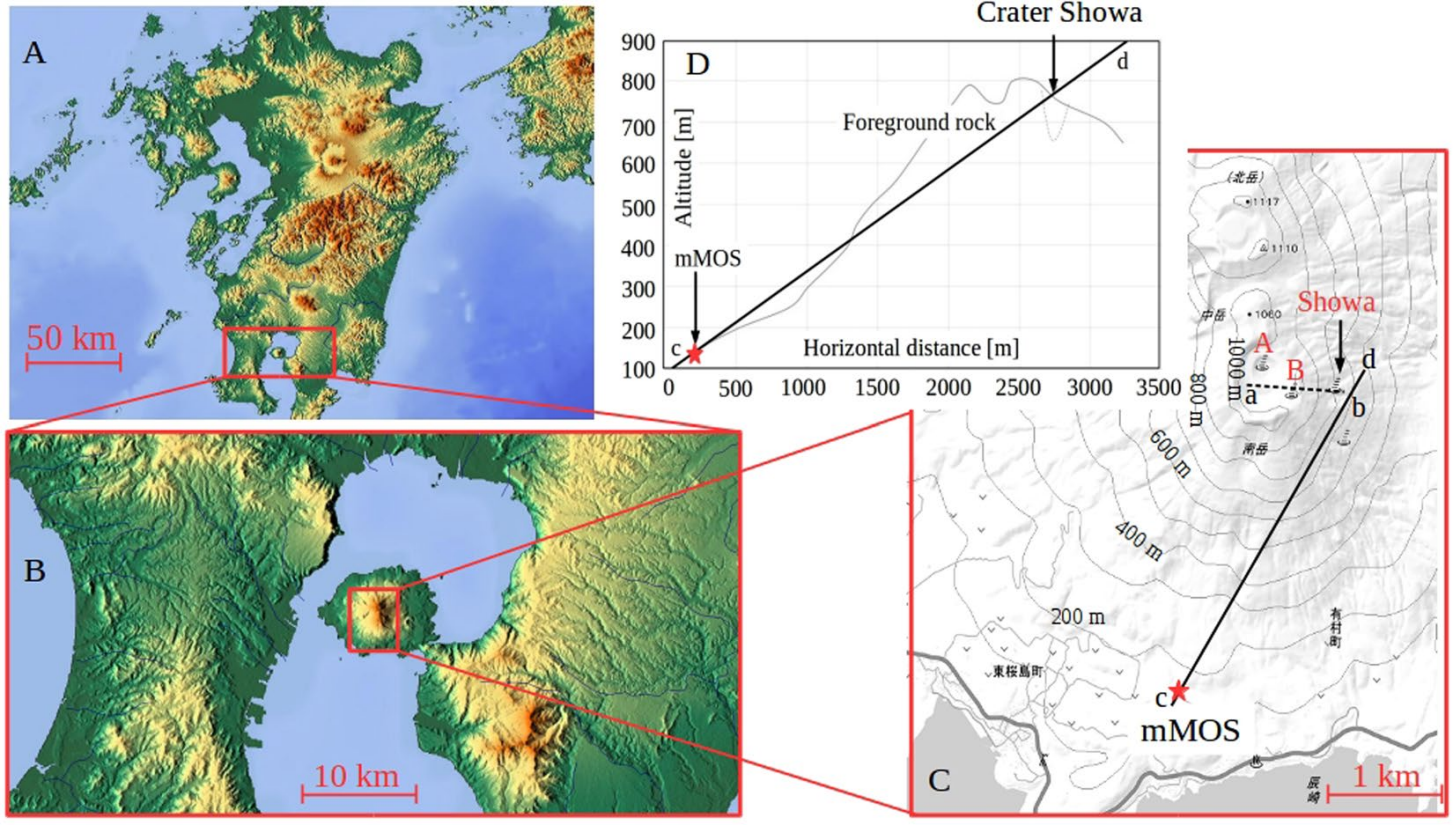
Schematic drawing of our muography experiment. (**A**) and (**B**) The location of the Sakurajima volcano in Kyushu, Japan (https://maps-for-free.com/)^19^. (**C**) The elevation map and the schematic of measurement site. The mMOS (*red star*) was installed in the South-West direction at the distance of 2.7 km, 2.6 km, and 2.8 km from the Craters A, B and Showa, respectively. The elevation map was created from the data of Geospatial Information Authority of Japan (http://www.gsi.go.jp/)^20^ and it was edited by the authors. (**D**) The cross-sectional view of Sakurajima volcano along the c-d line in (**C**).

The mMOS was installed at the Sakurajima volcano, Kyushu, Japan. Figure 1A,B,C and D respectively shows the location of the measurement site^19^, the topographic map of the measurement site and an example of the cross sectional view of Minami-dake, Sakurajima volcano. The elevation of the measurement site was above 140 m above sea level (ASL). The measurement was performed from the January of 2017 to July of 2017, and the data collected during 157 days were used to present analysis. The Sakurajima volcano is an andesitic volcano located in southern Kyushu, Japan and one of the most active volcanoes in Japan. Three active craters, called Craters A, B, and Showa are located at the top of Minami-dake, and Craters A and B are alternatively called the Crater Minami-dake. The elevation map in Fig. 1C was extracted from the data collected by the Geospatial Information Authority of Japan (GSI)^20^. The most active crater is the Showa crater located 500 m east of the summit. The shape of the Showa crater has been changed due to more than 2000 eruptions that took place during 2013, 2014 and 2015. The style of eruption is characterised by the recurrent Vulcanian eruptions, and a significant number of eruptions had occurred in Minami-dake Crater since 1955 but the main eruptive activity shifted from the Crater Minami-dake to Crater Showa after 2006. The main motivation of the present measurement was to image the fine geometrical structure underneath the Craters Minami-dake and Showa. The observation system (*red star*) was installed in the South-West direction at the distance of 2.7 km, 2.6 km, and 2.8 km from the Craters A, B and Showa, respectively. It was directed towards the Showa crater. The resulting image resolutions around the Craters A, B and Showa were expected to be between 7.4 × 7.4 m^2^ and 7.6 × 7.6 m^2^.

### Tracking system for high-definition and low-noise muography

**Figure 2:**
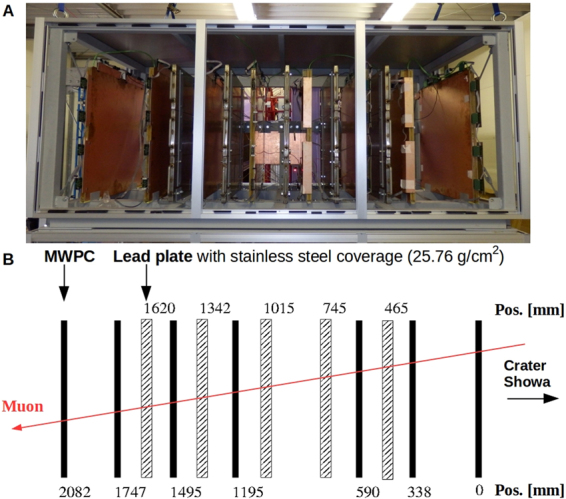
The MWPC-based Muographic Observation System (mMOS). (**A**) The photograph of mMOS from side view. **(B)** The schematic view of mMOS which consists of seven multi-wire proportional chambers and five lead shielding plates with the thickness of 2 cm each.

The multi-wire-proportional-chamber-based Muography Observation System (mMOS) was developed for imaging the internal structure of meter to kilometer-sized objects. It consists of seven multi-wire proportional chamber (MWPC) detectors with the size of 80 × 80 cm^2^ and five sheets of radiation shield made of lead plate. Each lead plate is concealed inside a stainless steel case in order to mechanically protect it and to prevent exposure of lead toxicity. Figure 2A and B respectively show the photograph and schematic draw of the mMOS from side view with the position of detectors and radiation shields. The fair position resolution of 4 mm in root-mean-square (RMS) of the MWPC detectors and the total tracking system length of 2,082 mm results in an overall angular resolution of 2 mrad. We used the inner five detectors for present analysis in order to increase the acceptance of observation system. The extended angle of view of the tracking system was ±517 mrad in both of vertical and horizontal directions. The positive and negative vertical angles correspond to forward directed and backward directed angles, respectively. The angular resolution of this detector arrangement was 2.7 mrad. Due to the fair position resolution the scattering of low-energy particles in the lead plates is measurable along the trajectory, and therefore these (<1 GeV) background particles can be removed from the recorded track sample. Such scattering is much reduced for those high energy muons which can cross the mountain material. Detailed technical description of the mMOS is provided in Method Section.

### Demonstration of high-definition muographic image construction

**Figure 3:**
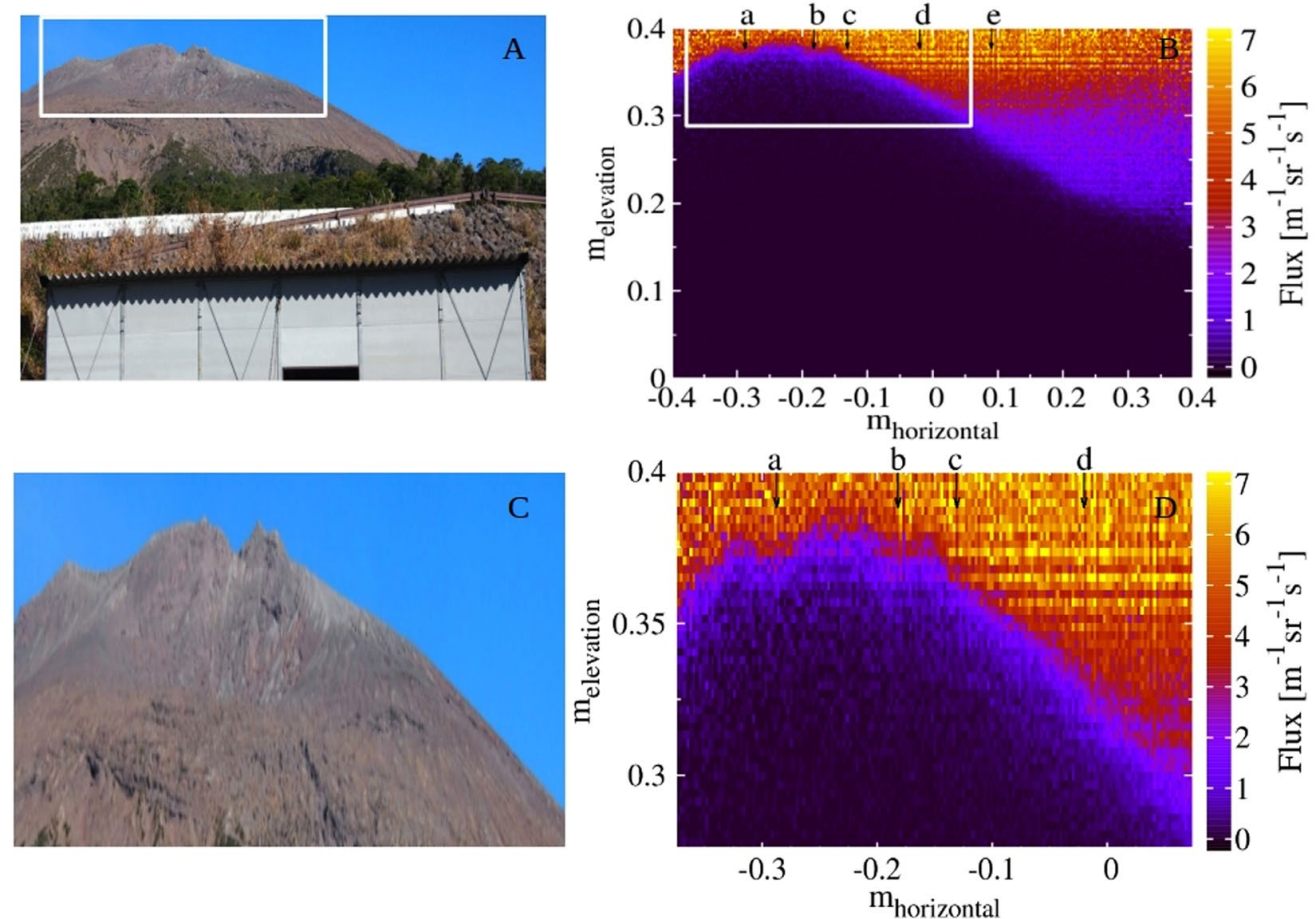
The first high-definition muographic images about the Sakurajima volcano. (**A**) and (**C**) The photographs of the Sakurajima volcano from the measurement site. (**B**) and (**D**) High-definition (tan(2.725 mrad) × tan(2.725 mrad)) muographic images of Sakurajima volcano well reproduce the shape of the volcano around the Craters A and B. White boxes in (**A**) and (**B**) indicate the regions which are magnified in (**C**) and (**D**).

To demonstrate the applicability of the mMOS to muography for high-definition imaging of volcanoes, the measured flux was compared to the photographs of Sakurajima volcano in selected angular ranges. The panels *A* and *C* of Fig. 3 show the photographs of the Sakurajima volcano. The corresponding fluxes are plotted as a function of tangent angles within different intervals on panels *B* and *D* with the definition of tan(2.725 mrad) × tan(2.725 mrad). The arrows show the location of the craters, namely the Crater A is located between **a** and **b**, the Crater B is located between **c** and **d** and Crater Showa is located between **d** and **e**. The muographic images qualitatively reproduces the outline of the mountain presented by the photographs.

### Imaging range of mMOS

**Figure 4:**
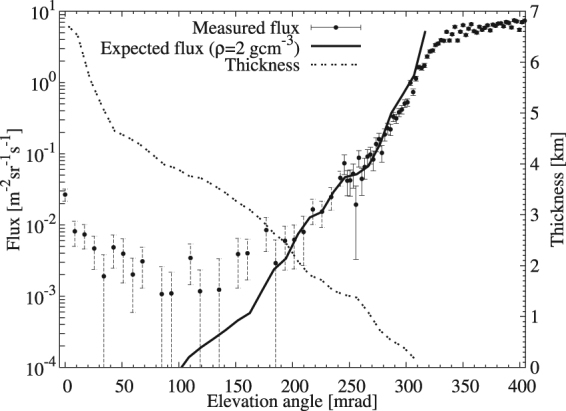
The comparison of measured and expected fluxes. The measured flux with 1σ error bars is plotted as the function of elevation angle within ±10.9 mrad horizontal angle range. A dotted and solid line respectively indicates the rock thickness and the flux calculated by assuming an average rock density of 2 gcm^−3^.

To confirm our measurement quality, the measured muon flux was compared to the expected flux calculated from the path length distribution of the volcano as a function of elevation angle in the *ϕ* = 0 horizontal direction. The expected muon flux was determined by integrating the zenith-angle dependent muon energy spectra based on the Modified Gaisser parametrisation^21^. The integration was performed over a range between a minimal energy that the muons could escape from a given thickness of rock with an average density of 2 gcm^−3^ (see in Method Section) and 20 TeV. The rock-thickness distribution from the detector point of view was calculated from the elevation data collected by GSI^20^. The calculation of the expected flux is detailed in Method Section. Figure 4 shows the measured (*black dots*) and expected (*black solid line*) flux in 21.8 × 10.9 mrad^2^ (*ϑ* ≤ 250 mrad) and 21.8 × 2.725 mrad^2^ (*ϑ* > 250 mrad) angular bins as a function of elevation angle. The measured flux was found in agreement with the expected flux within 1 errors above the elevation angle of 180 mrad. At lower elevation angles, the background effect is more enhanced and thus the measured flux was higher than the expected value. At the elevation angle lower than 180 mrad the path-length of muons across the volcano (*dashed black line*) exceeds 2.5 km (approximately 5,000 meter-water-equivalent). The deviation between the measured flux and the modeled flux at higher elevations is, on the other hand, caused by a difficulty in estimation of an effect from the local topography near the mMOS (See the little mound of the road shown in the Photograph Fig. 3A).

### The first high-definition muographs of the Sakurajima volcano

**Figure 5:**
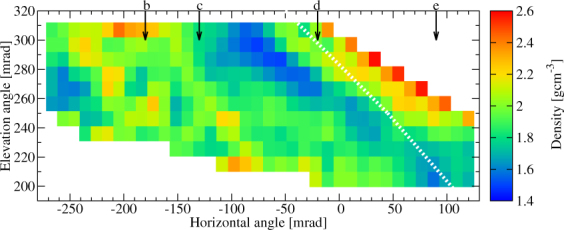
Muograph of Sakurajima volcano with the definition of 10.9 × 10.9 mrad^2^. A dotted line indicates the cross section of Sakurajima volcano along the a-b line in Fig. 1C.

**Figure 6:**
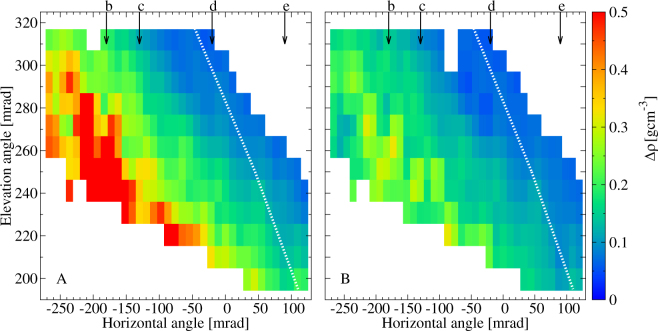
The errors of density with the definition of 10.9 × 10.9 mrad^2^. The lower (**A**) and upper (**B**) errors of density were calculated for the 1σ errors of the measured flux. The dotted lines indicates the cross section of Sakurajima volcano along the a-b line in Fig. 1C.

**Figure 7:**
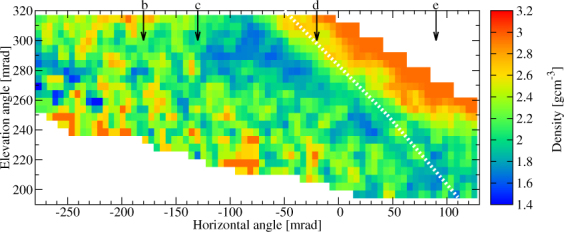
High-definition muograph with the pixel size of 5.45 × 5.45 mrad^2^. A dotted line indicates the cross section of Sakurajima volcano along the a-b line in Fig. 1C.

**Figure 8:**
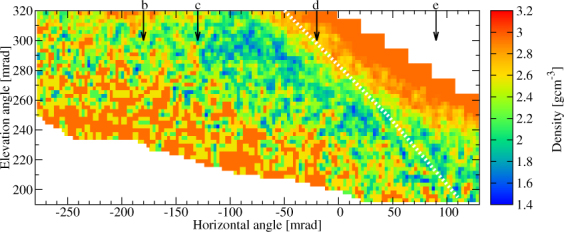
High-definition muograph with the pixel size of 2.725 × 2.725 mrad^2^. A dotted line indicates the cross section of Sakurajima volcano along the a-b line in Fig. 1C.

The measured flux was compared to the expected flux to determine the density distributions around the Craters B and Showa. The detailed procedure is presented in Method Section. The expected flux was modeled by using the topographic map created in 2013 but the actual size of the Crater Showa has been expanded since then. Therefore, a lower density will be imaged in the direction towards Crater Showa. Figure 5 shows the extracted average density in 10.9 × 10.9 mrad^2^ angular bins as the function of azimuth and elevation angles (ϕ, *ϑ*). The corresponding lower and upper density errors were also calculated for ±1σ errors of the measured flux. The lower and upper errors of the extracted average density are respectively shown in Fig. 6A and B. Figure 5 shows a low-density region with a density of (1.6 ± 0.15) gcm^−3^ underneath Crater B and inside Crater Showa while the density of other regions ranges between 1.8 gcm^−3^ and 2.2 gcm^−3^. The highest density (~2.6 gcm^−3^) derived along the upper edge of the image is due to difficulty in estimating the actual path length of the muons near the surface. The reason why a low density region appears also underneath the Crater B is probably because the crater depth on the topographic map is different from the actual one. Figures 7 and 8 show the higher-definition average density maps with the definition of 5.45 × 5.45 mrad^2^ and 2.725 × 2.725 mrad^2^. In Figs 5, 6, 7 and 8, the *white dotted line* shows the cross section of Sakurajima volcano along the a-b line presented in Fig. 1C and the arrows show the location of the craters as also shown in Fig. 3.

## Discussion

The sizes of the muographically imaged three small peaks at the top of Minami-dake (Fig. 3D) are 40 m, 20 m, and 30 m from the West to the East, and these peaks are respectively located at a distance of 2.43 km, 2.35 km and 2.40 km from the mMOS observation site, indicating that the angular resolution of the mMOS is achieved to be a few mrad. The smearing caused by the multiple scattering of penetrated muons has been investigated in GEANT4^22^ simulation framework for different thickness regions of volcano with realistic muon spectra and measurement arrangement (see in Method Section). The multiple scattering was defined as the angle between the actual and the expected arrival angle, and it includes the scattering in the volcano and in the detector material. The systematic effect caused by the multiple scattering of penetrated muons resulted in 4 mrad around the ridge of the volcano and 1.25 mrad−1.5 mrad around the investigated crater regions. The observed high-definition muographic images demonstrated the potential of the mMOS for high-definition imaging of interior of active volcanoes, especially for manifesting the presence of conduits with the diameter of few meters (10 m–50 m).

As can be seen in Fig. 4, the background rate is suppressed to less than 10^−2^ m^−2^ sr^−1^ s^−1^ by mMOS. This result demonstrated that the current mMOS was applicable to imaging the volcano up to the thickness of about 5,000 meter-water-equivalent. In order to image deeper regions, further reduction in the background rate will be necessary but this is out of scope of this report, and therefore we focus on the result imaged above an elevation angle of 200 mrad.

The low-density regions (blue patches seen in Figs 5, 7 and 8) that are located underneath Crater A (*ϕ* < −230 mrad, 250 mrad <*ϑ* < 280 mrad), Crater B (−130 mrad < *ϕ* < −20 mrad, *ϑ* > 260 mrad) and Crater Showa (−20 mrad < *ϕ* < 90 mrad, 200 mrad < *ϑ* < 250 mrad) could be the uppermost part of the magma pathway or the bottom part of these craters. The higher density region above the dotted line in Figs 5, 7 and 8 comes from the foreground rock effect shown in Fig. 1D. We now construct a picture of these three craters in order to interpret our muographic image and arrive at an estimate of their geometrical structures.

### Crater A

The elevation of the floor of Crater A is indicated to be 900 m ASL by the topographic map. No eruption has occurred at Crater A since 2013, and therefore we can assume that the crater depth has not changed since the time when the topographic map produced in 2013. Since the distance between the measurement site and the center of Crater A is 2.7 km, the elevation angle to the bottom of the Crater A is 280 mrad. However, the bottom edge of the low density region in the direction of Crater A is located at 240 mrad. This discrepancy is reflected by the actual depth of Crater A that was measured to be 600 m ASL in 2001.

### Crater B

The elevation of the floor of Crater B is indicated to be ~900 m ASL by the topographic map but it is not clear. Since the distance between the measurement site and the center of Crater B is 2.6 km, the elevation angle to the bottom of the Crater B is supposed to be ~300 mrad. However, the current image shows the bottom of the low density region in the direction of Crater B is located at an elevation angle of 250 mrad. On the other hand, it was reported that the depth of Crater B was measured to be 600 m ASL in 2001 but it became 650–680 m ASL due to recurrent crater wall collapses at sometime in the past. Although the current situation is different, the low-density region observed underneath Crater A could represent this discrepancy also. The higher density region indicated at an elevation angle of 220 and 240 mrad underneath Crater B might reflect degassed high-density magma, but it is statistically indistinguishable with the current data quality. We anticipate this question will be answered with the future upgraded mMOS.

### Crater Showa

The current elevation of the floor of Crater Showa is not known, and the topographic map we used for estimating the expected flux were produced in 2013, i. e. before the crater size was expanded. Therefore, the low-density region we observed in Figs 5, 7 and 8 simply reflects the newly expanded Crater Showa after 2013 eruptions. The bottom edge of the low-density region is located at least below an elevation angle of 200 mrad, indicating the bottom of the crater is located at an elevation of lower than 700 m ASL. Alternatively, this low density region might be reflected by an upper part of the magma pathway. As shown in Fig. 5, the density measured underneath Craters A and B was almost the same as the surrounding rock. This result is in contrast to the low density region that was observed underneath the crater of Asama volcano^6^. It is probably because fall back deposits filled the magma pathways underneath Craters A and B.

From this image, it was confirmed that current mMOS can be applied to monitoring the evolution of the shape of the crater of Sakurajima during the eruption without approaching there. In conclusion, the capability of mMOS to perform high-definition and low-noise muography was demonstrated. The methodology for producing the high-definition muographic images in Sakurajima volcano has been established.

## Methods

### Apparatus

The MWPC-based Muography Observation System is a modular tracking detector which consists of seven light-weight (~10 kg per layer) MWPCs and five lead (~150 kg per layer) shielding plates. The stack of the seven MWPCs provides spatial and directional information about the trajectory of detected particles. Three types of wires are applied in the MWPCs: 22 *μ*m-thick and 25 *μ*m-thick gold-plated tungsten wires are used as anode wires to achieve gas amplification, with 100 *μ*m thick field-shaping wires in between. Perpendicularly to the anode wires, 100 *μ*m-thick copper wires are used as signal pick-up wires, so that a single detector layer provides two-dimensional information^16^. The distances between the anode wires are 12 mm, resulting in a detector layer which is segmented into 64 × 64 pixels with the size of 12 × 12 mm^2^ each. The position resolution of the novel type of MWPC is below 4 mm^16^. This fair position resolution and the tracking system length of 2,082 mm provide the angular resolution of about 2 mrad (2.7 mrad for the inner five tracking layers) which value is excellent for muography. Environmental friendly and cost efficient Ar-CO_2_ gas mixture (Ar:80, CO_2_:20) flows continuously across the mMOS with the moderate flow rate of about 1–2 liters per hour. On the anode wires, a voltage of +1750 V is applied to achieve reasonable tracking efficiency of above 90% for each detector layer. The detailed description of the MWPCs about their structure, operation and performance has been presented in ref.^16^.

Five 2-cm-thick lead shielding plates coated with 4 mm-thick-stainless steel (with the density-length of 25.76 gcm^−^^2^ per plate) are applied between the MWPCs to absorb or deflect the low-energy background particles. These particles are mostly charged soft particles, hadrons and low-energy muons which arrive from the direction of the target of interest as a result of interactions without having penetrated it^23,24^. The energy of background particles was found below typically 1 GeV^23^. The expected scattering angle of 1 GeV particles across the 10-cm-thick lead shielding is rougly 60 mrad with Gaussian approximation which value is well above the angular resolution of the mMOS of 2.7 mrad.

The Data Acquisition (DAQ) and front-end electronics (FEEs) have been presented in more details in refs^16,25–27^ and here only a brief description is provided. The DAQ is controlled by a Raspberry Pi^28^ (RPI) computer. Trigger functions and data routing from the FEEs-s are implemented on a custom design board. The data acquisition is triggered by any three-fold coincidence of six MWPCs, which ensures that the trigger efficiency is practically 100%. The trigger signals are provided by the common signal of the sense wires. The FEEs amplify and discriminate the analog signals from the individual field wires and pick-up wires with low power CMOS integrated circuits. During data readout the trigger is blocked, which results in the dead time of about 100 *μ*s for the tracking system. The digitised data is zero-suppressed and collected into an ASCII file and stored on an Secure Digital card together with timing and trigger data, as well as with the analogue signal amplitudes of MWPC detectors. The typical size of the compressed data is as low as 0.1 kbyte per event. Remote detector control and data access can be performed via Virtual Private Network. The mMOS is supplied by a +12 V DC power supply adapter which can be connected to rechargable batteries or local electricity network. A power converter supplies the RPI, the DAQ boards and FEEs with +5 V DC as well as the high-voltage unit. The total power consumption of the mMOS is less than 10 W.

The MWPCs, the shielding plates and the DAQ are installed into an iron stand which is closed by plexi-glass walls (see in Fig. 2). Thus the system is isolated from highly humid environment of the measurement site in case of prolonged rainy weather. The installation time of the mMOS is typically between 4 hours and 6 hours for two or three persons.

**Figure 9:**
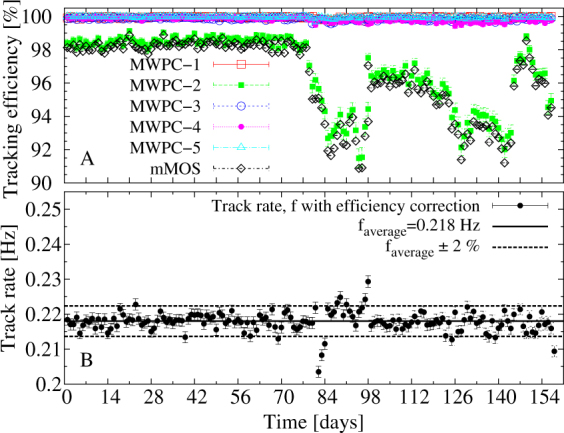
The operational stability of the mMOS. (**A**) The variation of tracking efficiency during the measurement campaign. (**B**) The efficiency corrected rate of tracks with ±1*σ* error bars varied around the average of 0.218 Hz within ±2%.

125.6 million triggered events have been recorded during the data taking time of 157 days. Figure 9 shows the tracking efficiency for the five MWPCs and for the mMOS (*A*), as well as the efficiency corrected track rate (*B*) as a function of time during the measurement campaign. The efficiency of tracking of particles with hits on all five detectors was found above 92% during the measurement campaign. (Note that the size of error bars are within the size of the points). The influence of temperature variation was negligible (<1%) on the tracking efficiency, because temperature compensation was implemented in the high-voltage unit^16^. The variation of tracking efficiency was caused by non-optimal gas pressure and flow after the gas bottle replacements. One of the MWPCs suffered more efficiency loss becasue the thicker anode wires were applied inside it^16^. The observed rate of tracks with hits on all five detectors was found around 0.218 Hz within ±2% during the data taking. Therefore, the mMOS operated reliably and provided data with excellent quality during the entire time of data taking. The regular maintenance of the tracking system is performed in every two months at the Sakurajima measurement site by two persons.

### Data analysis method

Event-by-event offline analysis has been performed on the collected data to determine the flux of muons. We used the inner five tracking layers for the data analysis. In the first step, the reconstruction of clusters and tracks was performed independently in both dimension^29^. Thereafter, the track projections were selected and the flux of muons was calculated.

The reconstruction was initiated by the determination of the clusters (continuous sequence of pick-up or field wires above discrimination threshold) with their position of centroids, size and number on each MWPC layer. The typical cluster sizes were two or three on the field shaping wires and from two to four on the pick-up wires because of their broad pad response function. Thereafter, track candidates were reconstructed with their main parameters (slope, intercept, and “goodness” of the fit) by a combinatorial algorithm and sorted by the“goodness” of the fit, *χ*^2^/*ndf*. The *χ*^2^ and *ndf* were defined as follows.

1 $${\chi }^{2}=\sum _{i\mathrm{=1}}^{{N}_{MWPC}}[\frac{{({x}_{cluster,i}-{x}_{track,i})}^{2}}{{{\rm{\sigma }}}_{MWPC,i}^{2}}],ndf={N}_{MWPC}-2,$$

where *x*_*cluster*,*i*_ was the reconstructed cluster centroids, *x*_*track*,*i*_ was the intersection of the fitted (*χ*^2^ minimising) straight track on the given detector layer, σ_*MWPC*,*i*_ ≈ σ_*MWPC*_ was the position resolution of the MWPCs, and *N*_*MWPC*_ was the number of MWPCs in the tracking system. We used the inner five tracking layers in present analysis, thus *N*_*MWPC*_ = 5.

The flux was calculated as follows.

2 $${\rm{d}}{F}_{meas}({m}_{h},{m}_{v})=\frac{{\rm{d}}N({m}_{h},{m}_{v})}{{\rm{d}}A({m}_{h},{m}_{v}){\rm{d}}{\rm{\Omega }}({m}_{h},{m}_{v}){\rm{d}}t},$$

where *m*_*h*_ and *m*_*v*_ were the slopes of track projections in horizontal and vertical directions, d*F*(*m*_*h*_,*m*_*v*_) is the flux, d*N*(*m*_*h*_,*m*_*v*_) was the number of detected tracks, d*A*(*m*_*h*_,*m*_*v*_) was the detector surface element, $${\rm{d}}{\rm{\Omega }}={\rm{d}}{m}_{h}{\rm{d}}{m}_{v}$$$${({m}_{h}^{2}+{m}_{v}^{2}+1)}^{\mathrm{3/2}}$$ was the differential solid angle in the (*m*_*h*_, *m*_*v*_) observation direction, as well as d*t* was the time of data taking. To determine d*N*(*m*_*h*_, *m*_*v*_), the following cuts were applied on the clusters and the tracks:

- The cuts on the number of cluster per MWPC, *N*_*clusters*_, and the length of clusters, *L*_*cluster*_ were applied to exclude the noisy events: *N*_*clusters*_ < 5 and *L*_*cluster*_ < 6.
- Single tracks with clusters on each of the five MWPCs were accepted.
- Tracks with the *χ*^2^/ndf < 2 were accepted as “straight” tracks.

Finally, the flux was re-binned into (*ϕ*, *ϑ*) bins, where *ϕ* is the azimuthal angle measured relatively to the orientation of the detector (clockwise directions have positive and anti-clockwise directions have negative horizontal angles, respectively) and *ϑ* is the elevation angle measured relatively to the horizon.

The tracking efficiency of the MWPCs was determined by a combinatorial algorithm. The examined MWPC was excluded and tracks were reconstructed on the other MWPCs. The tracks were extrapolated to the investigated MWPC; and the presence of clusters are examined within the fiducial region (±5σ_*MWPC*_) of extrapolated coordinate. The tracking efficiency of the MWPCs, *ε*_*i*_, *i* = 1, ..., 5, was given by the ratio of the number of found clusters and the number of extrapolations. The tracking efficiency of mMOS to detect tracks with five clusters using the inner five detectors – as we used mMOS in present study, *ε* was given by the following expression: *ε* = *ε*_1_*ε*_2_*ε*_3_*ε*_4_*ε*_5_.

### Analytical method

**Figure 10:**
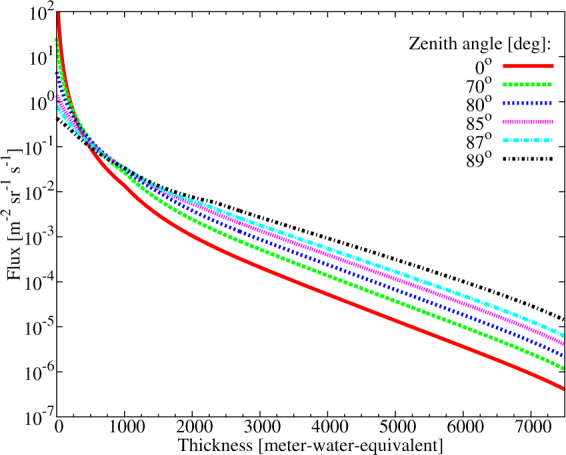
The attenuation of the muon flux across rock, given in meter-water-equivalent, for different zenith angles.

The attenuation of muon flux across the volcano measures the density-length (average density × thickness) of the volcano. Figure 10 shows the muon flux as a function of rock thickness in meter-water-equivalent units for different zenith angles. The density-length distribution of the volcano can be determined via the comparison of the expected flux, *F*_*calc*_ for various densities of the volcano to the measured flux, *F*_*meas*_ in various (*ϕ*, *ϑ*) bins. Thereafter, average density can be extracted along the path lines with the knowledge of the thickness of the volcano.

The expected flux, *F*_*calc*_(*θ*), of muons equals with the integral of the zenith angle dependent energy spectra, *f*_*diff*_(*E*, *θ*) from the minimum energy, *E*_*min*_ which is necessary for muons to penetrate the target of interest:

3 $${F}_{calc}(\theta )={\int }_{{E}_{min}}^{\infty }{f}_{diff}(E,\theta ){\rm{d}}E.$$

The path length across of the Sakurajima volcano from the detector point of view was calculated in 10.9 × 10.9 mrad^2^ bins using digital elevation map based on data collected by Geospatial Information Authority of Japan^20^. The measured map was improved by linear interpolation to achieve high-definition images. The minimum energy, *E*_*min*_(*θ*) was deduced from density-length using the continuous slowing down approximation range for Si0_2_^30^.

**Figure 11:**
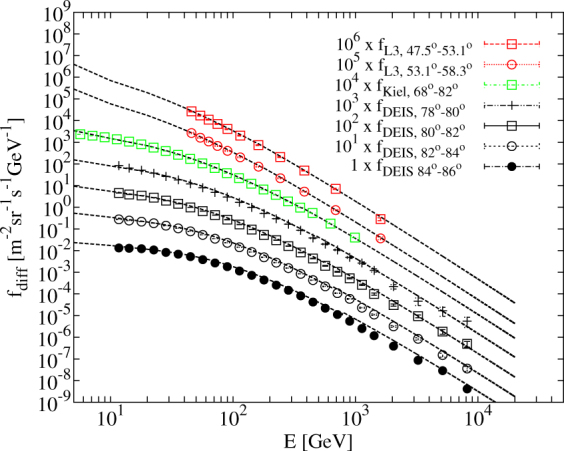
Comparison of muon spectra parametrised with Modified Gaisser model^21^ to the experimental data^32,33^.

The Modified Gaisser parametrisation^21^ was chosen to describe the spectra of muons and calculate the expected flux of muons. This parametrisation extends the so called Gaisser parametrisation^31^ to larger zenith angles (*θ* > 70°) and low-energy (E < 100/cos(*θ*) GeV) regions of the spectra. The original parametrisation of the spectra is described as follows.

4 $${f}_{diff}(E,\theta ){{\rm{cm}}}^{-2}\,{{\rm{sr}}}^{-1}\,{{\rm{s}}}^{-1}=A\,{E}^{-\gamma }[\frac{1}{1+(\frac{{a}_{0}E^{\prime} }{{\varepsilon }_{\pi }})\,\cos (\theta \text{'})}+\frac{B}{1+(\frac{{a}_{1}E^{\prime} }{{\varepsilon }_{K}})\cos (\theta ^{\prime} )}+{r}_{c}],$$

where the adjustable parameters are the factor *A*, spectrum index *γ*, *E* and *E*′ denotes the energy of muons on the surface of Earth and on the upper atmosphere, *θ* and *θ*′ denotes the zenith angle on the surface of Earth and on the upper atmosphere, *B* is the so-called balance factor which determines the branching ratios for the parent mesons, as well as *a*_0_ and *a*_1_ coefficients. The Gaisser parametrisation is the following:

*E* = *E*′, *θ* = *θ*′, *A* = 0.14, *B* = 0.054, *γ* = 2.7, *ε*_*π*_ = 115 GeV, *ε*_*K*_ = 850 GeV, *a*_0_ = *a*_1_ = 1.1 and *r*_*c*_ = 0^31^. This parametrisation describes the experimental data in the energy range of *E* > 100/cos(*θ*′). The modified parametrisation extends Equation (4) in the energy range of 1/cos(*θ*′) < *E* ≤ 100/cos(*θ*′) as follows^21^.

5 $$\begin{array}{rcl}E^{\prime} =E+{\rm{\Delta }},{\rm{\Delta }} & = & 2.06\times {10}^{-3}[\frac{950}{\cos (\theta )}-90],A=1.1{[\frac{90\sqrt{\cos (\theta )+0.001}}{1030}]}^{\mathrm{4.5/(}E\cos (\theta ^{\prime} ))},\\ \cos (\theta ^{\prime} ) & = & \sqrt{\frac{{\cos }^{2}(\theta )+{p}_{1}^{2}+{p}_{2}\,\cos \,{(\theta )}^{{p}_{3}}+{p}_{4}\,\cos \,{(\theta )}^{{p}_{4}}}{1+{p}_{1}^{2}+{p}_{2}+{p}_{4}}},{r}_{c}={10}^{-4},\end{array}$$

where Δ is the mean energy loss of muons in the atmosphere which should be take into account in the low-energy regions of spectra, the *A* is modified due to the nuclear enhancement of multiplicity and decay probability of muons; *θ*′ is expressed with constant parameters of *p*_1_ = 0.102573, *p*_2_ = 0.068287, *p*_3_ = 0.958633, *p*_4_ = 0.0407253, and *p*_5_ = 0.817285; as well as *r*_*c*_ is the ratio of prompt muons to pions. In the energy region of *E* ≤ 1/cos(*θ*′), the *E* → (3*E* + 7sec(*θ*′))/10 modification is done to better describe the experimental data. Figure 11 shows that the spectra parametrised using Modified Gaisser model (*black lines*) fits well onto the experimental data^32,33^ (*dots*) and the Gaisser model was implemented correctly in our calculations. The detailed description of the Modified Gaisser parametrisation is provided in ref.^21^.

**Figure 12:**
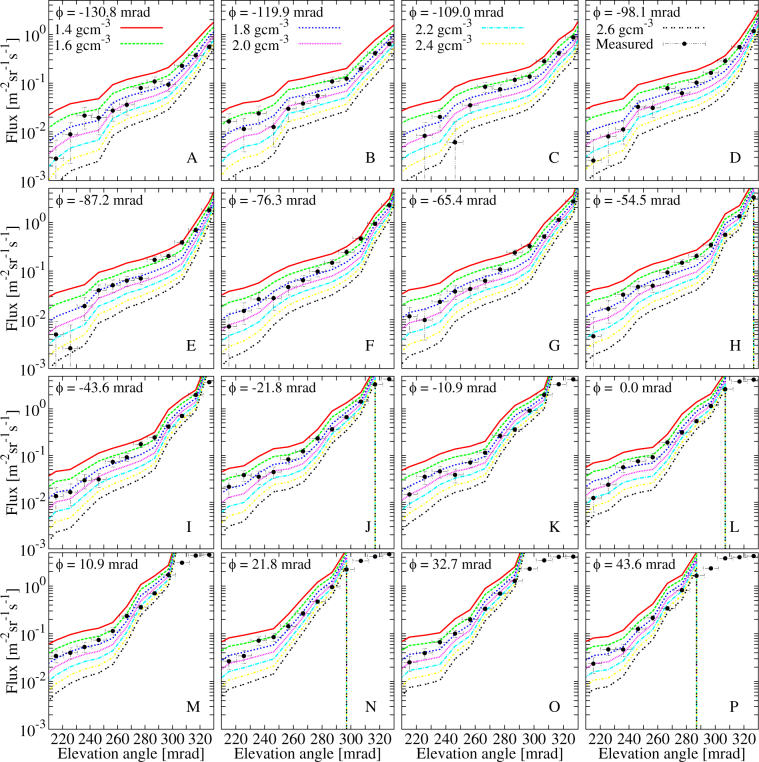
The measured flux slices (dots) with 1σ error bars and the modeled flux (lines) are plotted at different horizontal angles.

After the parametrisation of muon spectra, the expected flux was determined via the integration of the muon spectra from the minimum energy up to 20 TeV applying trapezoid integration method in each (*ϕ*, *θ*) bins. The expected muon flux was calculated for different densities in each angular bin and compared to the measured one. Figure 12 shows the comparison of the measured flux (*black dots with* ±1σ *error bars*) to the expected flux calculated for different average densities (*lines*) as a function of elevation angle for different azimuth angles between the Craters B and Showa. The comparison of flux slices hints the lower density region which is located between the elevation angles from 270 mrad to 310 mrad and between the azimuth angles from −130.8 mrad to −54.5 mrad (A-H), and from −43.6 mrad (I-P) it moves gradually to lower elevation angles with the increase of azimuth angle, as it is presented above in Figs 5, 7 and 8.

The density values were selected so that the difference of the measured and expected fluxes be minimal. The density errors were extracted as a function of *ϕ* and *ϑ* using the same procedure, except that instead of the 1σ errors of the measured fluxes, Δ*F*_*meas*_ the *F*_*meas*_ ± Δ*F*_*meas*_ was used for the comparison and later the difference of the extracted densities gave the density error, Δ*ρ*.

### Simulation method

The effect of the multiple scattering of penetrated muons on the muographic images has been investigated with GEANT4^22^ simulation for three different regions of the Sakurajima volcano: (A) close to the ridge at the zenith angle of 70° where the total thickness is roughly L ≈ 200 meter-standard-rock-equivalent (msre), (B) at the zenith angle of 75° where L ≈ 1000 msre, and (C) at the zenith angle of 79° where L ≈ 2000 msre.

**Figure 13:**
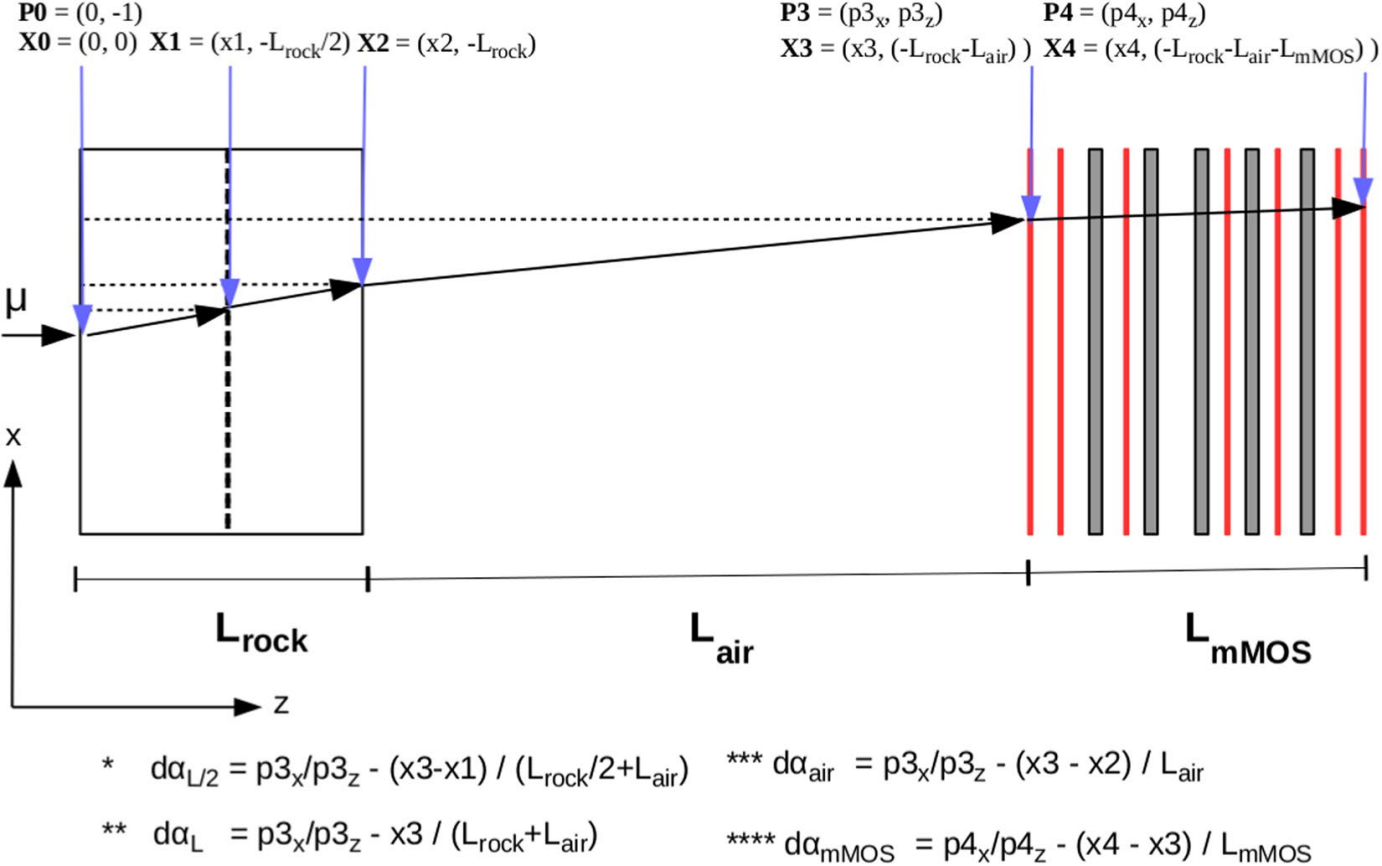
Shematic drawing of Monte Carlo simulation applied for the investigation of smearing effect of the multiple scattering of penetrated muons on the muographic images.

Figure 13 shows the geometric arrangement of the simulations in which L-thick rock wall, 3000 m air and the mMOS were implemented. The *red* plates show the detectors made from 2.5 mm-thick printed-circuit-board and the *gray rectangles* show the 2 cm-thick lead absorbers. The mMOS was implemented with the same geometric arrangement as it was applied at the Sakurajima volcano.

The muons were injected across a L-thick rock wall with the energy distribution parametrised by the Modified Gaisser model. The standard physics list with electromagnetic processes was applied in the simulation framework. The position vectors and momentum direction vectors of the generated muons were recorded along the trajectory of muons. Scattering angles were calculated from the middle of rock wall (d*α*_L/2_), from the other side of the rock wall (d*α*_L_), for the air (d*α*_air_) and inside the mMOS (d*α*_mMOS_). For example, d*α*_L_ defines the scattering angle of muon after the L-thick rock and after the 3000 m air, which is expressed by the difference between the its actual direction (p3x/p3z) and its expected direction (x3/(L_rock_ + L_air_)) on the first detector layer.

**Figure 14:**
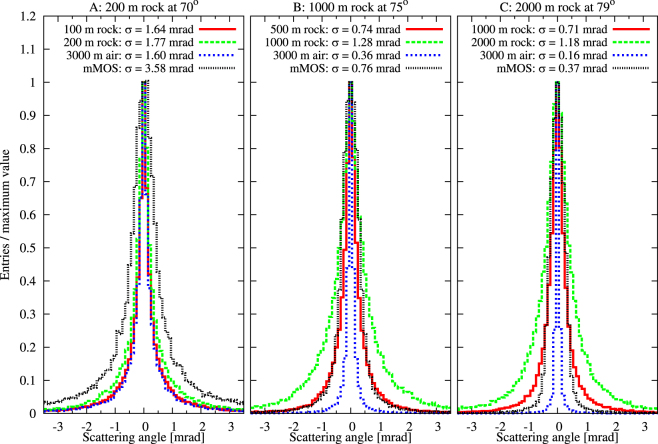
The distribution of simulated scattering angles of penetrated muons are plotted for the shallow part of the volcano (**A**) and for the crater regions (**B** and **C**).

Figure 14 shows the distribution of scattering angles and the corresponding RMS values of distributions for the middle 98 % of the entries after the half thickness (*red*), total thickness (*green*), air (*blue*), mMOS (*black*) for the 200 msre (A), 1000 msre (B) and 2000 msre (C), respectively. The effect of multiple scattering before the mMOS and in the mMOS is ${{\rm{\sigma }}}_{{\rm{MS}}}=\sqrt{{{\rm{\sigma }}}_{{\rm{L}}}^{2}+{{\rm{\sigma }}}_{{\rm{mMOS}}}^{2}}$, which results in 4 mrad for 200 msre, 1.5 mrad for 1000 msre, and 1.25 mrad at 2000 msre.

### Data availability statement

The datasets generated during and/or analysed during the current study are available from the corresponding author on reasonable request.
